# Time to abandon routine urine analysis prior to Bacillus Calmette–Guérin administration in asymptomatic patients

**DOI:** 10.1002/bco2.70117

**Published:** 2025-12-11

**Authors:** Antti Nummi, Pertti Nurminen, Olli Kesti, Mikael Högerman, Otto Ettala, Peter J. Boström, Antti Kaipia, Jukka Sairanen, Riikka Järvinen

**Affiliations:** ^1^ Department of Urology Helsinki University and Helsinki University Hospital Helsinki Finland; ^2^ Department of Urology Turku University Hospital Turku Finland; ^3^ Department of Urology Tampere University Hospital Tampere Finland

**Keywords:** adverse effects, asymptomatic bacteriuria, Bacillus Calmette–Guérin, BCG infection, complications, non‐muscle‐invasive bladder cancer

## Abstract

**Objective:**

This study aims to determine whether asymptomatic bacteriuria (ABU) increases the risk of infective complications requiring hospitalisation in patients undergoing Bacillus Calmette‐Guérin (BCG) instillations and consequently evaluate the need to screen asymptomatic patients for bacteriuria prior to BCG administration.

**Subjects/patients and methods:**

We analysed retrospectively all patients who received ≥1 BCG instillations as treatment of NMIBC in Helsinki University Hospital and Turku University Hospital during 2009–2018. Patients submitted urine specimens 1–7 days prior to every BCG instillation. Urine culture results and possible antibiotic prophylaxis prior to BCG administration were recorded. ABU was classified as having a positive urine culture but no dysuria or fever. All hospital admissions because of urinary tract infections and other BCG‐related adverse effects within 2 weeks of BCG administration were recorded.

**Results:**

We analysed 802 patients and 12 968 BCG instillations. ABU was recorded prior to 2428 (19%) instillations among which antibiotics were used in 527 (22%). Hospital admission was recorded after 9 (0,3%) and 39 (0,4%) instillations in the ABU and in the sterile urine groups, respectively (*P* = 0.9). Antibiotic prophylaxis did not affect the hospital admission rate (*P* = 0.2).

**Conclusion:**

BCG instillation with ABU is safe, and the results do not support routine screening of asymptomatic patients for bacteriuria prior to intravesical BCG immunotherapy.

## 1 INTRODUCTION

Asymptomatic bacteriuria (ABU) is common in patients with non‐muscle‐invasive bladder cancer (NMIBC) and does not usually require treatment.^1^, ^2^ Intravesical immunotherapy with Bacillus Calmette–Guérin (BCG) is the standard treatment in patients with high‐risk NMIBC.^3^, ^4^ Significant local and systemic adverse effects of BCG therapy are widely known, and concurrent urinary tract infection (UTI) is regarded as a risk factor.^5^, ^6^, ^7^

Serious adverse effects associated with BCG instillations may occur if the drug is absorbed systemically. Bacterial cystitis may impair the urothelial barrier, facilitating hematogenous dissemination of BCG.^6^, ^8^ However, it is suggested that ABU does not significantly increase the risk of infective complications during BCG therapy and it is not considered an absolute contraindication for intravesical BCG administration.^3^, ^9^, ^10^ Previous studies on ABU mainly use a single urine sample before the BCG induction period, potentially underestimating the total number of positive urine culture episodes during BCG therapy.^9^, ^10^

Current guidelines from the European Association of Urology (EAU) make no recommendations regarding the routine screening of urine for bacteriuria prior to BCG administration.^3^ Urine analysis is commonly performed prior to every BCG instillation, with many urologists preferring sterile urine or using prophylactic antibiotics to prevent complications.^11^ However, the evidence supporting this approach is limited. Positive urine culture in otherwise asymptomatic patients appears to be one of the most common reasons for delaying BCG instillations, potentially reducing treatment efficacy.^12^, ^13^ Additionally, antimicrobial treatment in asymptomatic patients may lead to the development of multidrug‐resistant bacterial strains.^14^

Routine urine analysis prior to every BCG instillation is a clinical practice in several hospitals in Finland, providing this study a large sample size throughout the BCG treatment. This study aimed to assess whether ABU increases the risk of infection‐related complications requiring hospitalisation and to evaluate the potential benefit of screening asymptomatic patients for bacteriuria before BCG instillations. Furthermore, we investigated whether the use of prophylactic antibiotics reduces the risk of symptomatic UTI requiring hospitalisation following BCG instillation in patients with ABU.

## 2 SUBJECTS/PATIENTS AND METHODS

### 2.1 Data sources and patient identification

This study was conducted at the University Hospitals of Helsinki and Turku, Finland. A retrospective search of institutional databases was performed using the intravesical BCG instillation operation code, including all patients who received ≥1 BCG instillation for NMIBC treatment between 2009 and 2018.

In case BCG treatment began before 2009, medical records were reviewed from the first BCG instillation of the regimen. For treatments extending beyond 2018, records were reviewed until the completion of BCG maintenance.

### 2.2 Characterisation of the patients

The following patient characteristics were assessed: sex, age, patient comorbidities using Charlson Comorbidity Index, smoking status, immunosuppressive state defined as the presence of haematological disorder, human immunodeficiency virus (HIV) infection, history of splenectomy, systemic immunosuppressive medication or daily prednisolone use ≥10 mg for any indication.

All patients with visible tumours underwent transurethral resection of bladder tumour (TUR‐BT) before BCG, and a biopsy was performed for those with carcinoma in situ. Tumour characteristics were recorded according to TNM and WHO 2004 classifications. Tumour size, multifocality, and NMIBC risk group using European Organisation for Research and Treatment of Cancer (EORTC) stratification were recorded.

### 2.3 BCG administration and bacteriuria

Patients underwent instillations of BCG through a urethral catheter by a urological nurse. After the induction course of 6 weekly BCG instillations, maintenance therapy was administered for 1–3 years according to SWOG (Southwest Oncology Group) or monthly instillation regimens. A clean‐catch voided urine specimen was submitted 1–7 days before each BCG instillation for bacteriological analysis. Urine cultures were recorded as no growth or bacteriuria (1000–100 000 organisms/mL, including mixed flora). Asymptomatic bacteriuria was classified as a positive urine culture without severe dysuria or fever. BCG instillation was postponed in case of symptomatic bacteriuria or suspected UTI. The use of antibiotic prophylaxis, complicated catheterisation, visible haematuria and elevated residual volume during BCG administration were recorded.

### 2.4 Characterisation of UTIs and BCG adverse effects

The symptoms of UTI were reviewed with patients before each BCG instillation by a urological nurse. All patients were instructed to contact the treating hospital in case of persistent symptoms of UTI (dysuria, urgency, fever ≥38°C, testicular pain/swelling and flank pain). As our primary endpoint, all hospital admissions because of UTIs or suspicions of UTI and other BCG‐related adverse effects within 2 weeks of BCG administration were recorded. The length of hospital stays, requirement of intensive care, symptoms of infection, urine and blood culture analysis, latency from the last BCG instillation and infection resolution were collected.

In Finland, BCG‐treated patients are advised to contact the tertiary care centre directly if any treatment‐related adverse events, including UTIs, occur. UTI was classified as having a positive urine culture with any symptom of UTI. All UTIs managed in an outpatient setting with patients contacting the treating hospital were also recorded as hospital admissions. Influenza‐like symptoms with a negative urine culture and without any other confirmed bacterial infections were recorded as BCG reactions. If antituberculosis treatment was given, the hospital admission was classified as a BCG infection.

### 2.5 Statistical analysis

Patient characteristics, results of urine analyses, and associations between presence and absence of bacteriuria with or without antibiotics for explanatory variables were summarised with descriptive statistics. Repeated measures were studied one by one with a generalised linear mixed‐effects model (GLMM).

The normality of variables was evaluated visually and tested with the Shapiro–Wilk test. The statistical significance level was set at 0.05 in all tests. The analyses were performed using RStudio (version 2022.10.31.83211) based on R (version 4.2.2) (RStudio, PBC, Boston, MA, USA).

## 3 RESULTS

We identified a total of 816 patients, who received ≥1 BCG instillations as treatment of NMIBC during 2009–2018. We excluded 14 patients because of missing clinical data. Finally, 802 patients and 12 968 BCG instillations were analysed.

Baseline characteristics are presented in Table 1. The majority (83%) of patients were male, and the median age was 73 years (IQR = 66–79). The median number of administrated BCG instillations was 17 (IQR = 12–21). Most patients (73%) received BCG instillations according to a monthly regimen. A total of 575 (72%) patients had at least one positive urine culture during the BCG regimen.

**TABLE 1:** Patient and tumour specific characteristics.

Variable		Total *n* = 802^1^
Age		73 (66,79)
Sex	Male	668 (83%)
	Female	134 (17%)
Smoking status	Never smoker	226 (32%)
	Ex smoker	286 (41%)
	Current smoker	188 (27%)
	Unknown	102
Charlson comorbidity index	2	388 (48%)
	3	202 (25%)
	≥ 4	212 (26%)
Immunosupression	No	769 (96%)
	Immunosupressive medication	16 (2%)
	Haematologic disease	14 (2%)
	Previous splenectomy	1 (0,1%)
	Unknown	2
Prior recurrence rate	Primary	573 (71%)
	Recurrent	229 (29%)
Tumour type	Ta	270 (34%)
	Cis	121 (15%)
	T1	406 (51%)
	T2–4	5 (0,6%)
Concomitant cis^2^	No	430 (54%)
	Yes	368 (46%)
Tumour grade	PUNLPM	4 (0,5%)
	Low grade	50 (6%)
	High grade	737 (93%)
	Unknown	11
Tumour multifocality	Solitary tumour	376 (48%)
	2–7	391 (49%)
	8≤	24 (3%)
Tumour size	< 1 cm	89 (11%)
	1–3 cm	448 (57%)
	> 3 cm	252 (32%)
	Unknown	13
EORTC^4^ risk group	Low risk	25 (3%)
	Intermediate risk	144 (18%)
	High risk	492 (61%)
	Very high risk	141 (18%)
Number of instillations		17 (12, 21)
Instillation regimen	Monthly	587 (73%)
	SWOG^3^	215 (27%)
Positive urine culture prior BCG instillation at any point of the BCG treatment	Yes	575 (72%)
	No	227 (28%)

^1^ Median (IQR); n (%).

^2^ Carcinoma in situ.

^3^ Southwest Oncology Group.

^4^ European Organisation for Research and Treatment of Cancer.

Characteristics of every BCG instillation, pre‐BCG urine analysis results and BCG postponements are presented in Table 2. Bacteriuria was recorded in 2428 (19%) urine cultures. *Staphylococcus* (32%), *Enterococcus* (28%), and *Escherichia coli* (14%) were the most common single pathogens. Antibiotic prophylaxis was given in 150 (1.4%) cases in the no growth group and 527 (22%) in the bacteriuria group (*p* < 0.001). There was no significant difference in the rate of BCG administration challenges between the groups (5.3% vs. 4.1%, respectively, *p* = 0,4). Instillation was postponed in 354 (2.7%) cases. Abnormal urine analysis was the most common reason for delay, accounting for 223 (63%) of postponed instillations and was more frequent in the bacteriuria group (*p* < 0.001). However, suspicion of symptomatic UTI was noted in only 68 (31%) of the postponed cases because of abnormal urine analysis.

**TABLE 2:** Characteristics of 12 968 BCG instillations administered to 802 patients with non‐muscle‐invasive bladder cancer. Presented by the presence or absence of bacteriuria on voided urine culture prior to each BCG administration.

Variable	Urine analysis result		Overall	*p*‐value^2^
	Sterile	Bacteriuria		
	*n* = 10 545^1^	*n* = 2428^1^	*n* = 12 968^1^	
Antibiotic prophylaxis	150 (1,4%)	527 (22%)	677 (5,3%)	<0.001
Single dose	97 (66%)	207 (40%)	304 (46%)	
≥ 3 days	49 (34%)	312 (60%)	361 (54%)	
Challenging BCG administration	552 (5,3%)	101 (4,1%)	653 (5,3%)	<0.001
Traumatic catheterisation	133 (24%)	19 (19%)	152 (23%)	
Visible haematuria	108 (19%)	10 (9,9%)	118 (18%)	
Elevated residual volume^3^	165 (30%)	66 (65%)	231 (35%)	
Other	150 (27%)	6 (5,9%)	156 (24%)	
Instillation postponed for any reason	128 (1,2%)	226 (9,3%)	354 (2,7%)	<0.001
Abnormal urine analysis^4^	10 (8%)	209 (92%)	219 (62%)	
No symptoms of UTI	1 (10%)	112 (54%)	113 (52%)	
Suspicion of symptomatic UTI	8 (80%)	60 (29%)	68 (31%)	
Unknown	1 (10%)	37 (18%)	38 (17%)	
BCG toxicity	56 (41%)	10 (4%)	66 (18%)	
Other	62 (48%)	7 (3%)	69 (19%)	

^1^ n (%).

^2^ Pearson's Chi‐squared test.

^3^ BCG emptied from the bladder with a catheter after 2 h.

^4^ Routine urine analysis prior BCG instillation without patient contacting hospital with symptoms of UTI.

Infective complications and other BCG‐related adverse effects requiring hospital admission are presented in Table 3. A total of 48 (0,4%) BCG instillations resulted in hospital admissions. We found no significant difference in hospital admissions between the groups with or without antibiotic prophylaxis (*p* = 0.2). Different levels of UTIs were the most common reason for hospitalisation, accounting for 26 (54%) of admissions. Hospital admissions because of BCG toxicity were recorded after 22 instillations, that is, 0.17% of all instillations, and accounted for 46% of all hospital admissions. Ten cases (21%) were classified as BCG infections requiring antituberculotic treatment. We found no significant difference in hospital admissions because of BCG toxicity between the groups. Intensive care was required in one case, and one severe BCG infection resulted in death.

**TABLE 3:** Adverse effects requiring hospital admission within 2 weeks of the last BCG administration. Presented by the presence or absence of bacteriuria on voided urine culture and if there was use of antibiotic prophylaxis prior to BCG administration. The numbers refer to BCG administrations.

Result of urinary culture and antibiotic prophylaxis prior BCG administration	Sterile	Sterile + ab^a^	Bacteriuria	Bacteriuria + ab^a^	Overall
	*n* = 10 376^b^	*n* = 148^b^	*n* = 1915^b^	*n* = 529^b^	*n* = 12 968^b^
Resulted in hospital admission	38 (0.4%)	1 (0.7%)	6 (0.3%)	3 (0.6%)	48 (0.4%)
Cystitis	1 (2.8%)	0 (0%)	1 (17%)	1 (33%)	3 (6.7%)
Epididymitis	2 (5.3%)	0 (0%)	0 (0%)	0 (0%)	2 (4.2%)
Pyelonephritis	14 (37%)	1 (100%)	2 (33%)	2 (67%)	19 (40%)
Urosepsis	0 (0%)	0 (0%)	1 (17%)	0 (0%)	1 (2.1%)
Fournier gangrene	1 (2.6%)	0 (0%)	0 (0%)	0 (0%)	1 (2.1%)
BCG reaction^c^	12 (32%)	0 (0%)	0 (0%)	0 (0%)	12 (25%)
BCG infection^d^	8 (21%)	0 (0%)	2 (33%)	0 (0%)	10 (21%)
Length of stay (d), *n* = 41	7 (4, 16)	4 (4, 4)	8 (6, 28)	6 (2, 9)	7 (4, 16)
Intensive care (d), *n* = 2	0	0	9 (8.5, 9.5)	0	0
Resulted in death	0	0	1 (17%)^e^	0	1 (2.1%)

^a^ Antibiotic prophylaxis.

^b^ *n* (%); median (IQR).

^c^ Influenza‐like symptoms without confirmed bacterial infection and without the use of antituberculotic treatment.

^d^ Infection warranting antituberculotic treatment.

^e^ Due to systemic BCG infection.

**FIGURE 1:**
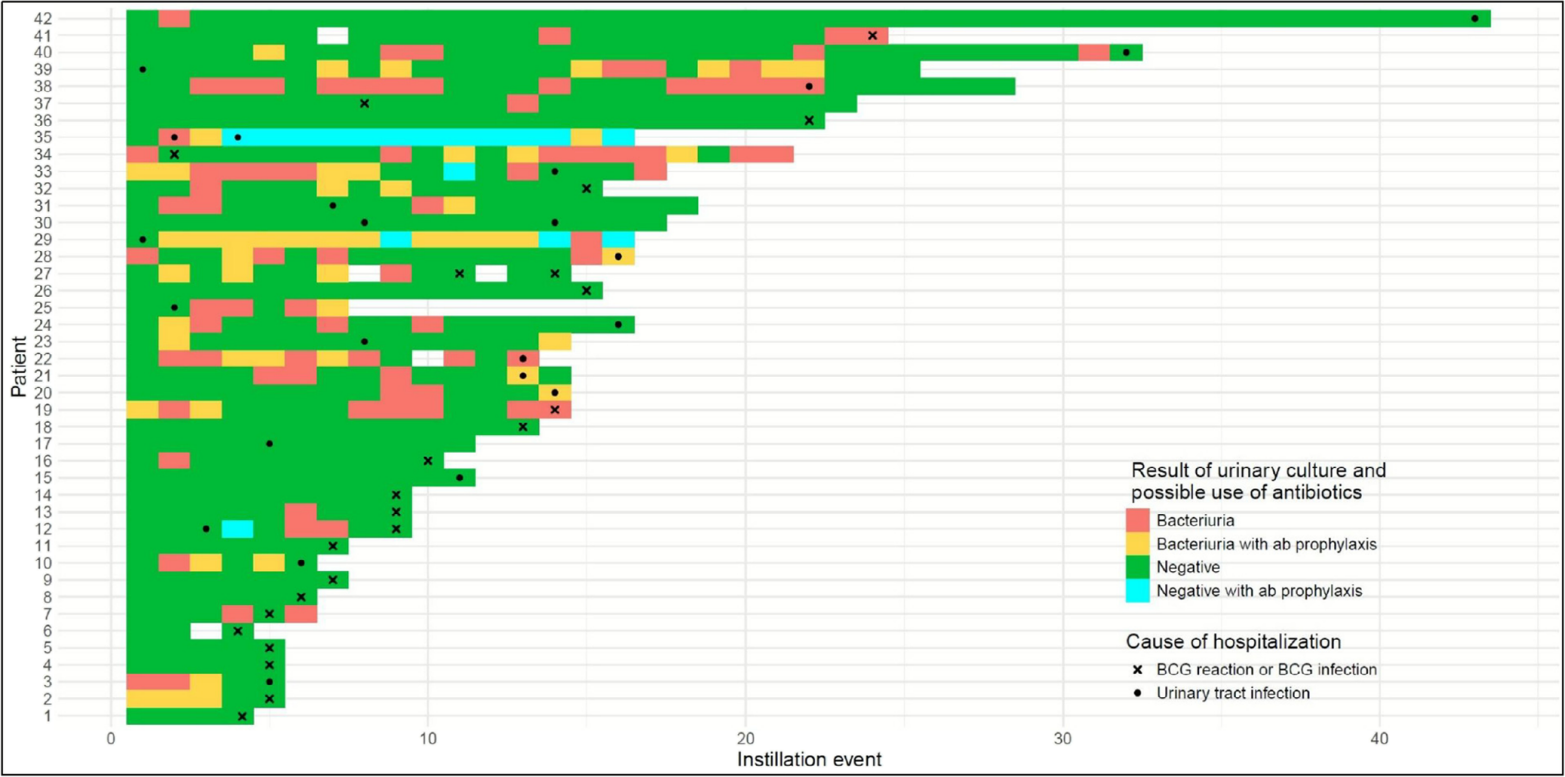
Swimmer plot of all 42 patients requiring hospital admission within 2 weeks of BCG administration, presented along with their pre‐BCG urinary culture results throughout the entire BCG regimen.

Figure 1 shows a swimmer plot of all patients requiring hospital admission, along with the results of previous urine cultures during the BCG regimen. A total of 42 patients required hospital treatment owing to infective complications or BCG‐related adverse effects, with six patients having multiple admissions. BCG treatment was discontinued as a result of hospitalisation in 27 patients of which 20 were because of BCG reaction or BCG infection.

## 4 DISCUSSION

In this study, we found no difference in the rate of infective complications or BCG adverse effects requiring hospitalisation between ABU and sterile urine during BCG instillation. We demonstrate that BCG immunotherapy is safe in patients with ABU, regardless of the result of the preceding urine culture. Antibiotic prophylaxis is not necessary to sterilise the urine prior to BCG administration. By consequence, our results do not support routine screening of asymptomatic patients for bacteriuria prior to BCG administration.

Few studies have compared the safety and efficacy of intravesical BCG immunotherapy with ABU and sterile urine.^9^, ^10^, ^15^, ^16^ Herr et al. reported a rate of ABU 25–53% in patients undergoing BCG therapy. In their studies, after the BCG induction period, 0.8–1.9% of patients developed febrile UTI in the ABU group and 0.6–3.6% in the sterile urine group, respectively.^9^, ^10^ These are results of one voided urine culture per patient at the beginning of BCG therapy. In our study, we report a similar rate of ABU (19%) based on all urine cultures, which included multiple urine cultures per patient throughout the BCG regimen. In total, 22 (2.7%) patients developed febrile UTI during the BCG regimen, with two patients experiencing more than one. Febrile UTI occurred after 19 (0.2%) and 7 (0.3%) BCG instillations in the sterile urine and bacteriuria groups, respectively, with no significant difference between the groups (*p* = 0.9). Our findings align with previous studies and EAU guidelines underlining that BCG therapy with ABU is as safe as with sterile urine.

It is unclear whether ABU affects BCG toxicity. Local and systemic adverse effects are common during BCG, causing 29–53% of treatment discontinuations.^5^, ^7^, ^17^ Brausi et al. reported that up to 70% of patients undergoing BCG experience local or systemic side effects, with 23% developing bacterial cystitis Similarly, Van der Meijden et al. observed a 26% bacterial cystitis rate in patients receiving BCG. Both studies also report chemical cystitis with negative urine cultures in 35% and 47% of patients. Given the high prevalence of ABU in NMIBC patients, it is challenging to distinguish whether local symptoms are because of BCG toxicity or bacteriuria. Mild to moderate irritative urinary symptoms are common after recent TUR and carcinoma in situ and may not indicate UTI. In our study, severe BCG adverse effects occurred after 20 (0.2%) instillations in the sterile urine group and after 1 (0.04%) instillation in the bacteriuria group, respectively. Of these, all but one hospital admission resulted in discontinuation of BCG therapy. Because our analysis focussed only on severe BCG adverse effects requiring hospitalisation, we cannot assess mild side effects or overall treatment tolerability. However, our results are in line with our hypothesis that ABU during BCG administration does not increase the risk of severe BCG adverse effects.

Current EAU guidelines make no recommendations regarding the routine screening of urine for bacteriuria before BCG administration. Still, urine analysis or culture is commonly performed prior to every BCG instillation. This strategy assumes that detection of ABU or pyuria could reduce the risk of systemic intravasation if BCG is avoided. Zhao et al. compared patients with and without pre‐BCG urine analysis and found no significant differences in UTI frequency or hospitalisations, suggesting that pyuria and bacteriuria on screening were poor predictors for UTI.^12^ In our study, no difference was found in the rate of infective complications requiring hospital admission between the sterile urine group (0.4%) and the bacteriuria group (0.3%) (*p* > 0.9). Positive urine culture in otherwise asymptomatic patients may lead to unnecessary delays in BCG instillations, potentially reducing treatment efficacy. Abnormal urine analysis in the routine pre‐BCG urine sample was the most common reason for delaying BCG instillation in our cohort, reported in 209 (8,6%) instillations in the bacteriuria group, of which only 60 (29%) reported irritative symptoms. Furthermore, routine urine analysis adds burden through frequent laboratory visits and increased costs. Our results support omitting routine screening of urine before BCG administration in asymptomatic patients.

Several studies have been conducted on the use of antibiotic prophylaxis with BCG therapy, yielding varying results regarding efficacy, tolerability and rate of infectious complications. Herr et al. demonstrated that induction BCG therapy can be safely performed without antibiotic prophylaxis even in patients with ABU, with symptomatic UTIs occurring at a low frequency (0.8–2.2%) in those with positive urine cultures.^9^, ^10^, ^16^, ^18^ However, the addition of fluoroquinolones before BCG instillation may decrease BCG toxicity and was even found to improve BCG efficacy.^19^, ^20^ However, in another retrospective study, concurrent antibiotic treatment with BCG therapy adversely affected the recurrence and progression of the disease.^21^ Although the EAU guidelines do not recommend using antibiotic prophylaxis with BCG in patients with ABU, many clinicians prefer sterile urine considering BCG administration as an invasive urological procedure.^3^ In our study, we found no significant difference in hospital admissions between the groups with or without antibiotic prophylaxis (*p* = 0.2). As the outbreak of multidrug‐resistant bacterial strains has become a major health concern, unnecessary antibiotics should be avoided to treat subclinical infections.^14^ Our results support that antibiotic prophylaxis prior to BCG administration is not necessary in patients with ABU.

The pathogenesis behind UTIs and severe BCG adverse effects share similarities.^6^, ^22^, ^23^ The bladder urothelium acts as a passive barrier preventing BCG and other pathogens from reaching the bloodstream. Breaches of this mucosal barrier, such as recent TUR or bacterial cystitis, have been identified as risk factors for both infectious complications and BCG adverse effects.^1^, ^6^, ^8^, ^24^ By consequence, symptomatic urinary tract infection is considered an absolute contraindication for BCG administration.^3^ In the case of ABU, the mucosal lining is not similarly disrupted, and clinical studies have shown that ABU may even protect against superinfecting symptomatic UTI pathogens.^25^ There is no direct toxic effect of BCG on tumour cells. Instead, it induces an intense local immune reaction that results in tumour destruction.^26^ Indeed, it has been demonstrated that intravesical BCG eradicated urinary tract pathogens in antibiotic‐naïve bladder tumour patients with ABU, suggesting that the same local immune reaction may protect against symptomatic UTI.^27^

The focus of this study was to investigate the risk of infective complications after a single BCG instillation. We do not report the effect of ABU on the overall tolerability or efficacy of BCG therapy, as individual patients often had both positive and negative urine cultures during the BCG regimen. Limitations of this study come with its retrospective setting. Without a predefined form for reporting adverse effects, we only captured severe complications requiring hospitalisation or other contact with the treating hospital. Our study relied on patients following the given instructions to contact the treating hospital if symptoms indicative of UTI occurred. This may underestimate the true incidence of milder, subclinical UTIs and other adverse effects. In addition, it has been reported that some local BCG infections may present after a long latency, making it difficult to retrospectively estimate which particular instillation led to BCG dissemination.^28^ A strength, however, is that our study presents a large patient and BCG instillation material with the largest published number of pre‐BCG urine cultures. Our data originated from two Finnish University Hospitals with similar patient material and clinical practice policies.

Our results support omitting routine screening of asymptomatic patients for bacteriuria prior to intravesical BCG immunotherapy. Asymptomatic bacteriuria is a common finding in a bladder cancer patient undergoing BCG therapy and does not require antibiotic prophylaxis. Administering intravesical BCG in patients with ABU is safe and does not increase the risk of developing severe UTI or BCG complications.

## AUTHOR CONTRIBUTIONS

Antti Nummi had full access to all data in the study and takes responsibility for the integrity of the data and the accuracy of the data analysis.

*Study concept and design*: Antti Nummi, Pertti Nurminen, Riikka Järvinen, Jukka Sairanen, Otto Ettala, Antti Kaipia and Peter J. Boström. *Acquisition of data*: Antti Nummi, Pertti Nurminen and Olli Kesti. *Analysis and interpretation of data*: Antti Nummi, Mikael Högerman, Pertti Nurminen, Jukka Sairanen and Riikka Järvinen. *Drafting of the manuscript*: Antti Nummi. *Critical revision of the manuscript for important intellectual content*: Pertti Nurminen, Riikka Järvinen, Jukka Sairanen, Otto Ettala, Antti Kaipia and Peter J. Boström. *Statistical analysis*: Mikael Högerman. *Obtaining funding*: None. *Administrative, technical, or material support*: None. *Supervision*: Riikka Järvinen and Jukka Sairanen. *Other*: None.

## CONFLICT OF INTEREST STATEMENT

The authors declare no conflicts of interest relevant to this work.
